# Is it time to switch to bivalirudin for ECMO anticoagulation?

**DOI:** 10.3389/fmed.2023.1237601

**Published:** 2023-08-21

**Authors:** Amir Navaei, Vadim Kostousov, Jun Teruya

**Affiliations:** ^1^Department of Pediatrics, Texas Children’s Hospital, Baylor College of Medicine, Houston, TX, United States; ^2^Department of Pathology & Immunology, Baylor College of Medicine, Houston, TX, United States; ^3^Department of Medicine, Texas Children’s Hospital, Baylor College of Medicine, Houston, TX, United States

**Keywords:** heparin, bivalirudin, review, ECMO, monitor

## Abstract

For decades, unfractionated heparin (hereafter, heparin) has been the primary anticoagulant used for extracorporeal membrane oxygenation (ECMO) support. More recently, however, bivalirudin, a direct thrombin inhibitor, has emerged as an alternative. This systematic review based on PRISMA guidelines, aims to summarize 16 comparative studies and 8 meta-analysis and review articles published from January, 2011 till May, 2023 which directly compares ECMO courses using heparin versus bivalirudin as the anticoagulant. While this comparison is complicated by the lack of a standardized definition of major bleeding or thrombosis, our overall findings suggest there is no statistical difference between heparin and bivalirudin in incidence of bleeding and thrombosis. That said, some studies found a statistical significance favoring bivalirudin in reducing major bleeding, thrombosis, and the need for transfusions. We also offer essential guidance for appropriately selecting an anticoagulant and monitoring its effect in ECMO settings.

## Introduction

Extracorporeal membrane oxygenation (ECMO) is widely used to support the heart and/or lungs, and the conditions for which it is indicated have recently expanded to include sepsis, extracorporeal cardiopulmonary resuscitation (ECPR), and coronavirus disease 2019 (COVID-19). It is also now used as a bridge to lung transplantation or implantation of a ventricular assist device (VAD) (1, 2). For many years, unfractionated heparin (hereafter, heparin) has been the primary anticoagulant used with ECMO. Here, we review the literature comparing bleeding, thrombosis, hospital course, and outcome among patients on ECMO using either heparin or bivalirudin as the anticoagulant, and we offer recommendations for the selection and monitoring of anticoagulants in this context.

### Choice of anticoagulant

Heparin is currently the primary anticoagulant used by the vast majority of institutions [94%, according to a recent survey (3)] in pediatric and adult ECMO management. Typically, once two units of red blood cells are in the blood reservoir, 200 units of heparin are added to the pediatric circuit and 1,000 units are added to the adult circuit. A bolus of heparin (50–100 units/kg) is also administered during cannulation (4) and, depending on the risk of bleeding, it is continuously infused post-cannulation. More recently, however, a small percentage of centers (6%) reported using bivalirudin as the primary anticoagulant for ECMO, 60% of which were primary neonatal and pediatric centers (3, 5).

### Heparin vs. bivalirudin

Heparin has several advantages: it is easy to use, relatively inexpensive, and in case of significant bleeding or overdose, it can be neutralized by administering protamine. On the other hand, heparin alone has no anticoagulant effect. To become an anticoagulant, it must form a complex with antithrombin (AT) and heparin cofactor II and release tissue factor pathway inhibitor (TFPI) from endothelial cells to express its entire anticoagulant action. AT plays the most important role in this process, and in 2014, the Extracorporeal Life Support Organization (ELSO) guidelines first suggested AT monitoring and replenishment during ECMO. However, the need to monitor and replenish AT levels, especially among newborns, remains controversial (6). In general, young children have lower physiologic concentrations of natural anticoagulants, including AT, and this is most true of children under 1 year of age. However, heparin, which is negatively charged, binds to more than 100 positively charged plasma proteins, including von Willebrand factor and C-reactive protein non-specifically (7, 8). When the patient on ECMO is in acute phase reaction, all acute phase reactant levels are increased, and overall heparin anticoagulant effect may be reduced. Due to its binding with many plasma proteins, the anticoagulant effect of heparin thus needs to be constantly monitored by activated partial thromboplastin time (aPTT) and/or anti-factor Xa assay (anti-Xa).

Bivalirudin, on the other hand, binds directly to thrombin, which is both circulating and clot-bound. Therefore, it does not require binding to other proteins to exhibit its anticoagulant effect. Approximately 20% of bivalirudin is cleared from the kidneys and the remainder is proteolyzed. While there are no reversal agents for bivalirudin, it has a much shorter half-life of 25 min in adults and 15 min in newborns, compared to heparin’s half-life of 90 min (9). Therefore, it is not a significant problem using bivalirudin in cases of active bleeding during ECMO support. A summary of this comparison can be found in Table 1. A growing number of studies have evaluated the use of bivalirudin versus heparin for ECMO.

**Table 1:** Heparin and bivalirudin comparison.

Drug	Heparin	Bivalirudin
Action	Binding to antithrombin and heparin cofactor II → anti-Xa and anti-IIa Release tissue factor pathway inhibitor	Binding to circulating and clot bound thrombin
Half-life	90 min	15–25 min
Clearance	Mainly reticulo-endothelial system (RES), some renal clearance	Mainly extravascular proteolysis, 20% renal clearance
Binding protein	>100 plasma proteins including antithrombin and heparin cofactor II	Only thrombin
Bioavailability	Poor	Good
Monitoring	aPTT and anti-Xa	aPTT, HPTT, dTT, ECT, ECA, anti-factor IIa
Antidote	Protamine (Heparin rebound may happen)	None

dTT, diluted thrombin time; HPTT, heparinase treated activated partial thromboplastin time; aPTT, activated partial thromboplastin time; ECT, ecarin clotting time; ECA, ecarin chromogenic assay; AT, antithrombin.

## Methods

**Figure 1:**
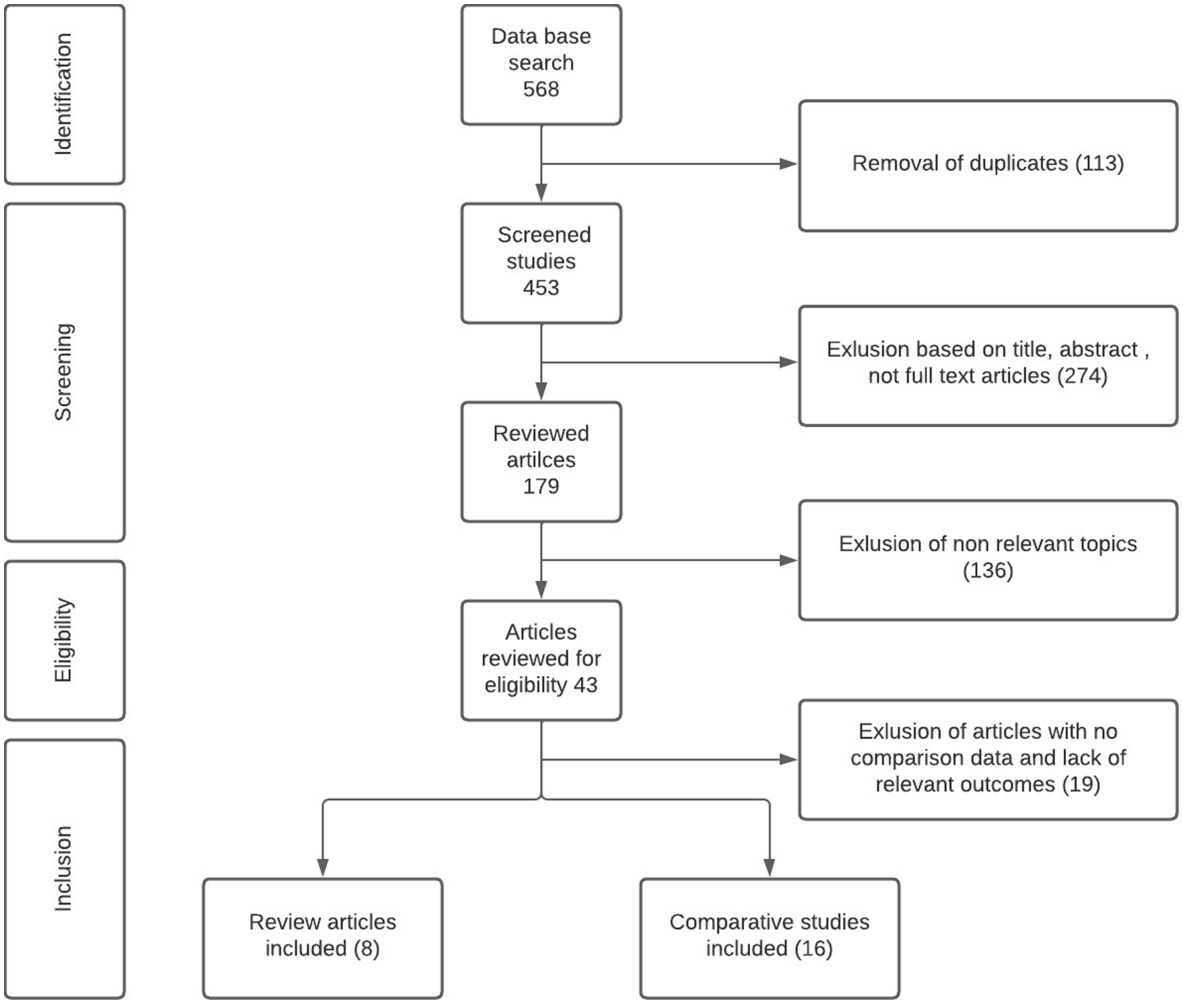
Flow diagram according to PRISMA guidelines.

We conducted a systematic review of the literature for anticoagulation comparison between bivalirudin and heparin in ECMO patients. Systematic review was performed according to Preferred Reporting Items for Systematic Reviews and Meta-Analyses (PRISMA) by using Embase, PubMed and Cochrane Libraries. Literature review performed by following keywords: (ECMO, extracorporeal membrane oxygenator, ECLS, extracorporeal) AND (heparin OR unfractionated heparin) AND (bivalirudin OR angiomax). Titles were screened by two independent reviewers. Full text articles were reviewed with following inclusion criteria: (1) including ECMO patients, (2) comparing bivalirudin and heparin, (3) have outcome measurements, and (4) January, 2011- May, 2023 (Figure 1).

In our literature review we identified 16 such studies, all of which were retrospective cohort studies published from 2011 to 2023. The majority included adult patients, while only 5 focused exclusively on pediatric patients. There is no universal scoring system for bleeding severity, so to compare these studies we rated bleeding severity using Bleeding Academic Research Consortium (BARC) scoring (10).

## Results

A total of 1,365 adult and 312 pediatric ECMO runs were collectively included in these studies, of which 558 adults and 116 pediatric patients received bivalirudin for anticoagulation (Tables 2A,B) (11–26). Six studies evaluated time to achieve therapeutic target with three showing significantly shorter time for bivalirudin and three finding no difference (12, 13, 17, 20, 22, 24). Seven studies assessed the percentage of aPTT being at therapeutic range, of which four (57%) showed bivalirudin having a significantly higher percentage of therapeutic laboratory targets versus heparin (12, 13, 20–24).

**Table 2A:** Review of studies comparing heparin and bivalirudin for ECMO anticoagulation. Comparison of heparin vs bivalirudin.

Author, year	Adults (Children)	VV or VA	Heparin	Bivalirudin	Thrombosis, %	Circuit Exchange, %	Major Bleed, %	Minor Bleed, %	ECMO Mortality, %	Transfusion reduction	Time to Target hours	TTR, %	Other findings
Tong et al., 2023 (11)	34	VV	20	14	35 vs 14, ns	—	30 vs 0, p=0.02	55 vs 57, ns	40 vs 7, p=0.033	OR 0.51, p=0.04 (RBC)	—	—	Platelet transfusion volume (0 vs 300 mL) and the incidence of major bleeding were significantly lower in bivalirudin group.
Uricchio et al., 2023 (12)	143	VA	89	54	0.1 vs 0.04 per day, p<0.001	—	49 vs 29, p=0.02	15 vs 13, ns	58 vs 74, p=0.04	OR 0.45, p=0.004 (RBC/day)	28 vs 21, p=0.001	40 vs 50, p<0.001	Bivalirudin was independently associated with an increased time to thrombosis. Patients receiving bivalirudin required less total RBC (p=0.04) and plasma administration (p=0.03).
Sheridan et al., 2022 (13)	150	VA	50	100	0 vs 1, ns	4 vs 4, ns	4 vs 6, ns	8 vs 4, ns	50 vs 57, ns	—	18 vs 2, p<0.001	33 vs 86, p<0.001	No difference was found in rates of bleeding between 2 groups. Bivalirudin yielded higher percent time in therapeutic range, faster time to therapeutic range and required fewer dose adjustments to maintain therapeutic range (2 vs. 11, P <0.001) compared to heparin.
Seelhammer et al., 2021 (14)	333 and (89)	VA 84%	223 and (65)	110 and (24)	17 vs 12, ns; (22 vs 13, ns)	5 vs 4, ns; (6 vs 13, ns)	—	—	53 vs 38, p=0.01 (57 vs 42, ns)	OR 0.28, p=0.02 (24 h, peds)	—	—	The reduced mortality in the adult bivalirudin group and reduction in the composite transfusion requirement in the first 24 hours was noted in the pediatric bivalirudin group.
Rivosecchi et al., 2021 (15)	295	VV	162	133	33 vs 17, p=0.003	—	41 vs 12, p<0.001	—	38 vs 27, p=0.054	OR 0.38, p<0.001 (RBC)	—	—	Overall decrease in the number of device thrombotic complications, significant increase in time to circuit thrombosis in bivalirudin group. Heparin was associated with significantly higher risk of clot formation. Significantly less volume of RBC, plasma and platelet transfusion and decreased number of major bleeding events in patients receiving bivalirudin.
Giuliano et al., 2021 (16)	144	VA 81%	131	13	0.05 vs 0.03 per day, ns	—	0.08 vs 0.03 per day, ns	—	62 vs 69, ns	OR 0.34 p<0.001 (RBC)	—	—	The rate of bleeding or thrombotic complications did not differ between heparin and bivalirudin, but patients on bivalirudin received significantly fewer blood transfusions (1.0 units of RBC/day vs 2.9 units/day on heparin; p<0.001).
Kaushik et al., 2021 (17)	(35)	VA 86%	27	8	—	11 vs 25, ns	44 vs 13, ns	4 vs 13, ns	26 vs 62, ns	—	12 vs 14.5, ns	44 vs 65, p=0.014	3/8 patients switched to bivalirudin due to concern for HIT and 1/8 for heparin resistance. Patients in the primary bivalirudin group had more stable aPTT values with only 9% of aPTT values having more than 30% variability from goal aPTT compared to 30% in the heparin group (p=0.003). Patients with primary bivalirudin use had a significantly lower dose requirement at 12 (p=0.003), 36 (p=0.007), and 48 (p=0.0002) hours compared to patients with secondary use of bivalirudin.
Rabinowitz et al. 2021 (18)	(67)	VA 61%	35	32	—	—	—	—	34 vs 37, ns	—	—	—	No correlation between anticoagulant dosing and any laboratory test variables in 37% of the heparin and 44% of the bivalirudin group.
Schill et al, 2020 (19)	(48)	VA 67%	34	14	21 vs 7, ns	20 vs 57, ns	12 vs 7, ns	—	27 vs 36, ns	OR 1.03, ns (RBC)	—	—	The bivalirudin group had longer support duration compared to the heparin (11 vs 3.3 days, p<0.001), but the freedom to first circuit intervention was longer in bivalirudin group (P = 0.02).
Machado et al., 2020 (20)	(32)	VA	14	18	6 vs 29, ns	35 vs 22, ns	11 vs 36, ns	—	43 vs 11, p=0.04	OR 0.4, ns (total)	13 vs 21, p=0.06	54 vs 57, ns	Mean percentage of time with therapeutic aPTT and ACT was not different. Phlebotomy- associated blood loss per ECMO hour was double in heparin 1.08 mL/hour compared to bivalirudin 0.51 mL/hour (p<0.001) as well as interventions to control bleeding.
Kaseer et al., 2020 (21)	52	VA 54%	33	19	33 vs 26, ns	27 vs 26, ns	18 vs 5, ns	—	45 vs 37, ns	—	—	50 vs 86,p=0.007	No difference in composite thrombotic or bleeding complications. Higher percent time to be in therapeutic aPTT range with bivalirudin.
Hamzah et al., 2020 (22)	(32)	VA 91%	16	16	19 vs 0, ns	6 vs 0, ns	75 vs 19; p=0.001	—	44 vs 38, ns	—	29 vs 11, p=0.001	—	Shorter time to therapeutic target time with bivalirudin. Less bleeding events per 10 days of ECMO support with bivalirudin (p=0.002). Lower rates of RBC and plasma transfusion with bivalirudin. Total cost of therapy less with bivalirudin (US $1184 vs. $494 per day; p=0.03)
Macielak et al., 2019 (23)	110	VA 67%	100	10	0.21 vs 0.09 per day; ns	—	0.22 vs 0.14 per day, ns	—	—	OR 1.17 (RBC)	—	38 vs 53, p<0.001	There were no differences in the rate of bleeding events. An increased number of adjustments to either anticoagulant was associated with a higher rate of bleeding events per day (p = 0.006).
Berei et al., 2017 (24)	72	VA 92%	28	44	25 vs 23, ns	—	25 vs 45, ns	25 vs 23, ns	32 vs 36, ns	—	56 vs 25, ns	83 vs 88, ns	No significant difference between heparin and bivalirudin for bleeding and thrombotic complications.
Pieri et al., 2014 (25)	20	VA 50%	10	10	30 vs 10, ns	—	40 vs 30, ns	20 vs 0, ns	50 vs 40, ns	—	—	—	No significant difference between heparin and bivalirudin for bleeding and thrombotic complications.
Ranucci et al., 2011 (26)	12 and (9)	VA	3 and (5)	9 and (4)	25 vs 8, ns	—	25 vs 0, ns	—	63 vs 31, ns	OR 0.6, ns (RBC)	—	—	No significant difference between heparin and bivalirudin for bleeding and thrombotic complications.

ns = not significant, TR = transfusion reduction, VV = veno-venous ECMO, VA = veno-arterial ECMO, RBC = red blood cells, TTR = therapeutic target range, HIT = heparin induced thrombocytopenia.

**Table 2B:** Number of publications with outcomes favoring bivalirudin or heparin (11–26).

	Favoring bivalirudin	Favoring heparin	No difference
Thrombosis	2	0	12
Major bleeding	4	0	10
Minor bleeding	0	0	6
Mortality	3	1	11
Transfusion reduction	5	0	4

Bleeding and thrombotic complications are the most clinically significant concerns associated with ECMO. A majority of the studies (13/15) evaluated major bleeding—i.e., bleeding associated with a hemoglobin drop of at least 2 g/dL, or a transfusion requirement of one or more doses of 10 mL/kg red blood cells (RBC) transfusion in a 24-h period. The bleeding included retroperitoneal, pulmonary, gastrointestinal, and central nervous system bleeding; bleeding that required surgical intervention; central cannulation site bleeding that required re-exploration; and fatal bleeding (type 3–5 according to BARC scale) (10). Overall, these studies suggest there is no significant difference between bivalirudin and heparin in the incidence of major bleeding (13, 16, 17, 19–21, 23–26). Four studies showed a significant decrease in the frequency of major bleeding in the bivalirudin group (11, 12, 15, 22). One study found a significant decrease in the number of laboratory tests in the bivalirudin group, which was associated with significantly lower blood loss. This latter finding is consistent with the fact that aPTT was more often at target, so use of bivalirudin required less frequent blood draws and adjustments (20). Two studies also showed that the bivalirudin group required significantly fewer RBC transfusions (15, 16). Thus, overall findings suggest that the risk of bleeding is the same with bivalirudin and heparin, but the former may have some benefit in reducing the need for phlebotomy and potentially decreasing the risk of bleeding.

Thrombotic complications can be a clinical marker of inadequate anticoagulation during an ECMO course, but they are challenging to assess retrospectively due to a lack of standardized reporting. The majority of studies (11/15) evaluating thrombotic complications of ECMO circuit reported no significant difference between the use of bivalirudin and heparin (11, 13, 14, 16, 19–25). Two studies showed significantly fewer ECMO-associated thrombotic events per day and an increased time to thrombosis among those receiving bivalirudin versus heparin (12, 15). One study showed a longer duration of freedom to circuit intervention (19) Thus, overall findings suggest that bivalirudin does not increase thrombotic complications in ECMO and may instead decrease them due to more stable anticoagulation.

One major concern about bivalirudin is that it costs more than heparin. Some studies— mainly in patients using a ventricular assist device (VAD)—evaluated the cost of each medication as well as the cost of associated laboratory tests and heparin’s additional requirement of AT replacement, especially in pediatric patients. While Hamza, et al. found a significantly lower overall cost of therapy with bivalirudin compared to heparin ($494 vs. $1,184 per day; *p* = 0.03) (24), other studies found that, overall, the costs are equal. When using bivalirudin, the cost of the drug itself accounted for a higher percentage of overall costs, whereas the larger percentage of heparin’s costs were attributable to laboratory diagnostic studies and AT replacement (27).

Once a clot is formed, heparin cannot inhibit clot-bound thrombin and slow down the process of clot propagation. In contrast, the relatively small molecular size of bivalirudin allows it to bind to both plasma-free thrombin and clot-bound thrombin. This is a potential advantage over both heparin and low molecular weight heparin (LMWH), as clot-bound thrombin remains active during amplification of the coagulation cascade (9).

In sum, bivalirudin’s potential benefits are that it: offers more stable anticoagulation; provides a faster response; requires less titration; and potentially decreases circuit clotting and major bleeding complications. Moreover, bivalirudin is no longer considered a novel anticoagulant as many centers, particularly adult hospitals, have become more familiar with it. Therefore, we suggest transitioning from heparin to bivalirudin as the primary agent for ECMO anticoagulation, keeping in mind that heparin may continue to be needed for new circuit priming and bolus administration until use of bivalirudin is established for these purposes.

### Heparin monitoring

In 90% of centers across the US, heparin monitoring is performed via anti-factor Xa (anti-Xa) activity assay. The common target level for thrombosis is 0.3—0.7 units/mL (7). (In our institution, we use the default target of 0.3–0.5 units/mL for ECMO). In 75% of centers, activated clotting time (ACT) is also used as an adjunct test, with 180–200 s the most common target but ranging from 180–240 s in centers using different ACT machines. Although aPTT and anti-Xa are recommended as the most reliable combination for monitoring heparin anticoagulation, the combination of ACT and anti-Xa is still most often (68%) used for this purpose (4). Viscoelastometric studies such as thromboelastography (TEG™) or rotational thromboelastometry (ROTEM™) are used in 41% of centers. Antithrombin level is now more frequently monitored, using targets that range widely from 60–100%. Institutions are improving in establishing protocols, but almost 50% still manage ECMO anticoagulation without a standard protocol, especially in hospitals with mixed adult/pediatric patient populations. Along with anti-Xa, aPTT is commonly targeted for heparin anticoagulation monitoring with a wide range of 60–100 s, depending upon patient age (4). aPTT results can be affected significantly by heparin contamination and underlying coagulopathy, such as disseminated intravascular coagulation (DIC) or dilutional coagulopathy, depending on whether the sample is drawn from a central line or directly from the ECMO circuit. Heparinase-treated aPTT (HPTT) helps to eliminate the heparin effect and reveal the underlying coagulable state, yet HPTT is still rarely used in most institutions due to its cost. It is also important to note that phospholipid-binding proteins such as lupus anticoagulant and C-reactive protein (CRP) may also prolong aPTT and HPTT (7, 28) and this effect must be considered when assessing aPTT and anti-Xa in gaging anticoagulation with heparin.

Anti-Xa assay is the most reliable test for measuring heparin’s effect because it directly measures heparin anticoagulant activity (29). There are various types of anti-Xa assays and the lack of standardization can cause a discrepancy in results. Anti-Xa assays that do not contain exogenous AT and rely solely on endogenous AT activity are preferred for evaluating heparin anticoagulation (30). They are helpful in cases of heparin resistance due to low AT levels or polymorphism of the binding site. Significantly elevated factor VIII may shorten aPTT and artificially present as heparin resistance. Therefore, the best practice is using both aPTT and anti Xa to target heparin’s effect (28).

### Bivalirudin monitoring

Several laboratory tests are available for monitoring the anticoagulant effect of bivalirudin, of which aPTT and ACT are the most commonly used. aPTT is the most common monitoring tool, with the standard target of 60–80 s (4). ACT is also used in some centers as an adjunct to aPTT (31).

Other coagulation assays include diluted thrombin time (dTT), chromogenic anti-factor IIa (anti-IIa) assay, ecarin clotting time (ECT), and the ecarin chromogenic assay (ECA) (9). Preanalytical errors such as heparin contamination can significantly affect aPTT, especially when the sample is collected through indwelling lines with a heparin lock. Therefore, HPTT should be used since heparinase is able to neutralize up to two units/mL of heparin in 500 μL of citrated plasma (32). The main disadvantages of using HPTT are its cost, incubation time, and lack of automatization; for these reasons, most centers do not use it as the standard test. Appropriately collecting samples from the ECMO circuit, not from an indwelling catheter, would decrease heparin contamination and is advised for institutions that do not have HPTT available. In our institution, we routinely use HPTT as a reflex test for possible heparin-contaminated aPTT samples screened by prolonged thrombin time and as part of a comprehensive ECMO panel.

Prolonged aPTT and HPTT may also be caused by other factors, such as lupus anticoagulant, high CRP, deficiency of intrinsic factors, or the presence of factor-specific inhibitors. Some studies therefore found that aPTT does not correlate well with bivalirudin level. Bivalirudin level measured by dTT has been reported to better correlate with bivalirudin administration dose (33, 34). Using dTT from spiked pooled normal plasma, *in vitro* studies reported concentrations anywhere from 0.2 to 1.5 μg/mL for bivalirudin, correlating with 1.5 to 2.5 times prolongation of normal aPTT values (33–35). Our institutional target range using dTT is 0.8 to 2.6 μg/mL as equivalent to 1.7–2.3 prolongation of upper normal aPTT value and is similar to previously described ranges—i.e., 0.5–2.5 μg/mL, as reported by Hasan, et al., using the same assays and reagents (36).

### Special considerations

ECMO cannulation requires intraoperative anticoagulation to prevent thrombotic complications due to hypercoagulable state secondary to endothelial cell injury, interruption of blood flow, and exposure to the cannula and foreign surfaces. The current practice is to administer 50–100 units/kg of heparin as a bolus dosing during cannulation with ACT monitoring intraoperatively. Bolus dosing of bivalirudin has been used mainly in cardiac catheterization and in interventional radiology procedures in adults. Due to the lack of pediatric data regarding bivalirudin bolus dosing, it is more difficult to standardize it in a pediatric population. The only contraindication for a heparin bolus would be suspicion of heparin-induced thrombocytopenia (HIT), which is uncommon in patients younger than 10 years old. Therefore, it is preferable to continue using a heparin bolus during ECMO cannulation.

Due to the ratio of circuit volume to total blood volume, pediatric patients—especially infants—are at higher risk of developing post-cannulation dilutional coagulopathy. This issue has been addressed by our institution and others by priming the circuit with plasma and RBC. Prior to starting bivalirudin, it is important to make sure dilutional coagulopathy is resolved and have a baseline aPTT level. In this way, appropriate anticoagulation can be achieved by targeting aPTT or HPTT. Also, aPTT can be prolonged due to lupus anticoagulant or C-reactive protein, so in patients with a high aPTT at baseline, it is important to target bivalirudin anticoagulation by targeting 1.5-2X increased in aPTT from the baseline.

Due to the short half-life of bivalirudin, its levels may be reduced in areas where blood is static. The ECMO circuit, which is different from cardiopulmonary bypass, does not contain a reservoir or areas of significant concern for stasis, so this risk is more likely in patients with anatomical etiologies for stasis, such as cardiac stunning, which may increase thrombotic complications. There is a paucity of data on this topic and each patient needs to be assessed individually. If prolonged stasis in a patient is of concern, heparin could be the preferred anticoagulation of choice. Clamping of cannulas during weaning trials from veno-arterial (VA) ECMO may potentially cause stagnation. Cannulas are unclamped periodically to prevent this outcome, but there is no standard methodology for doing so. (In our institution we unclamp every five minutes to avoid stagnation.) If prolonged stasis is a concern, practitioners may consider administering a heparin bolus of 25 units/kg prior to the clamping trial. Also, when bivalirudin is administered to the circuit during clamping trials, the patient may not receive adequate anticoagulation and therefore may need additional bivalirudin during them.

Bivalirudin clearance depends on renal function but is independent of dose and gender. Approximately 20% of the unchanged drug is cleared via the kidney, and the remainder presumably undergoes proteolysis intracellularly (37). Dialysis and increased urine output will reduce bivalirudin’s effect. In a retrospective multicenter study, it was reported that in pediatric ECMO population a decreased bivalirudin dose requirement of about 28% among patients with estimated glomerular filtration rate (eGFR) of >60, 30–60 and < 30 mL/min/1.73m^2^. Also, patients with lower eGFR had longer duration of supratherapeutic anticoagulation targets, higher bleeding tendency and transfusion of blood product requirement, close monitoring and dose adjustment (38). In the setting of accidental overdose or active bleeding that requires reversal of the bivalirudin effect, typically discontinuation of bivalirudin infusion is sufficient due to short half life, however, administration of plasma, prothrombin complex concentrate and hemodialysis can be considered in severe cases.

Severe acute respiratory syndrome coronavirus 2 (SARS-COV2) infection showed unique coagulation challenges during COVID-19 pandemic. Patients with severe SARS-COV2 infection demonstrated diffuse endothelial injury, micro and macrovascular thrombosis. Heparin was the standard anticoagulation of choice for thromboprophylaxis in COVID-19 infection. In severe SARS-COV2 patients with acute respiratory distress syndrome (ARDS) many patients required ECMO support for prolonged durations. In a retrospective case control study patients with SARS-COV2 ARDS on ECMO required higher doses of bivalirudin to achieve therapeutic aPTT targets compared to non COVID 19 group. However, there was no difference between the bleeding and thrombotic complications among two groups (39).

Prevalence of obesity among children is increasing in the United States. Obese patients not only have higher risk of thrombosis but also require special considerations for drug dosing. In a retrospective study conducted in patients with high body mass index (BMI) who had HIT, bivalirudin dosing based on total body weight was found to have most accurate predictor of achieving aPTT goal (40).

Management of ECMO anticoagulation in patients with underlying hepatic synthetic dysfunction, shock liver and DIC in the setting of septic shock can be challenging. Safety of using bivalirudin in adult patients with hepatic and renal dysfunction has been demonstrated in the literature (41). However, there is scarcity of pediatric and adult ECMO data. For management of anticoagulation with bivalirudin in patients with underlying coagulopathy, dTT and viscoelastometric studies would be instrumental. Consultation with coagulation expert and individual based risk assessment is essential in this population.

Coronary artery disease (CAD) is more common in adult population, however, coronary and other vascular stent placements occasionally required in pediatric patient as well. Using antiplatelet agents along with anticoagulants such as heparin or bivalirudin are the routine management to prevent thrombosis of the stent. ECMO patients due to decreased platelet function and acquired von Willebrand syndrome, potentially have a higher risk of bleeding when antiplatelet agents are utilized. A retrospective single center study showed no difference in bleeding complications in patients on VA ECMO who received dual antiplatelet therapy along with heparin for anticoagulation compared to heparin monotherapy (42). In as sub study of a randomized control trial conducted in Sweden, in patients with ST elevated myocardial infarction (STEMI) who are not on ECMO and undergoing primary percutaneous coronary intervention (PCI) and treated with aspirin and potent P2Y12-inhibitors, anticoagulation with bivalirudin was not superior to heparin regarding the composite end point of all-cause death, myocardial infarction, or major bleeding (43). A recent published randomized controlled trial in patients with STEMI undergoing primary PCI who had not received previous glycoprotein IIb/IIIa inhibitors compared bivalirudin with a post-PCI high-dose infusion vs. heparin monotherapy, showed significantly reduced the 30-day composite rate of all-cause mortality or BARC types 3–5 major bleeding in bivalirudin group (44). Due to scarcity of data in using antiplatelet agents along with bivalirudin vs. heparin in ECMO patients, decision needs to be made based on individual risk factors.

### Institutional experience and proposed ECMO anticoagulation protocol with bivalirudin as the primary anticoagulant

#### Historical aspect and current practice

Bivalirudin was introduced to our practice in 2016. Initially utilized mainly for ECMO and VAD anticoagulation when encountered heparin resistance. Currently, in our institution, the pediatric intensive care unit (PICU) and cardiac intensive care unit (CICU) both use bivalirudin as the primary anticoagulant for all ECMO runs. Our neonatal intensive care unit (NICU) continues to use heparin as the primary anticoagulant, while bivalirudin is the secondary agent and used mainly for heparin resistance. However, based on the growing experience of our primary care providers and the cost–benefit ratio, we are considering transitioning to bivalirudin as the primary anticoagulant for all ECMO runs, including for newborns in the NICU. Multimodal ECMO coagulation monitoring with standard coagulation assays and ROTEM has been utilized to manage complex ECMO coagulation management. Initially, HPTT has been the main targeted coagulation assay for bivalirudin management and recently added dTT alongside HPTT to improve accuracy.

### Proposed anticoagulation protocol

#### Pre-cannulation

Before ECMO cannulation, it is important to evaluate baseline coagulable state to assess underlying coagulopathy, which will guide the choice of circuit prime and transfusion requirements. A comprehensive multimodal approach is advised, including complete blood count (CBC), prothrombin time (PT)/international normalized ratio (INR), aPTT/HPTT, fibrinogen, D-dimer, ACT, AT, plasma hemoglobin (PHb) and TEG or ROTEM. The baseline evaluation should include von Willebrand factor (VWF) activity, VWF antigen, and VWF activity/VWF antigen ratio (45).

#### Circuit prime

Heparin remains the main anticoagulant of choice for priming the circuit when an RBC prime is used, mainly because more practitioners have experience with it and due to the lack of standardized dosing for other anticoagulants. Following ELSO recommendations, RBC priming should be used for the majority of circuits if time allows. In an emergency setting, crystalloids can be used for patients weighing >30 kg. As there are no standardized recommendations for circuit priming, it is important to consider adding plasma to the priming if the circuit volume is >50% of the total blood volume.

#### Heparin bolus during cannulation

Heparin remains the preferred anticoagulant during cannulation given: the negligible incidence of HIT in pediatric patients; institutions’ significant experience with the effects of heparin in cardiopulmonary bypass; and its reversibility with protamine. Depending on bleeding risk, a bolus of 50–100 units/kg should be administered during cannulation.

#### Post-cannulation

After cannulation, it is important to repeat coagulation tests, including PT/INR, aPTT/HPTT, D-dimer, fibrinogen, hemoglobin, and platelet count. Prompt correction of coagulopathy with plasma, cryoprecipitate, and platelet transfusions is essential to decrease bleeding complications.

**Figure 2:**
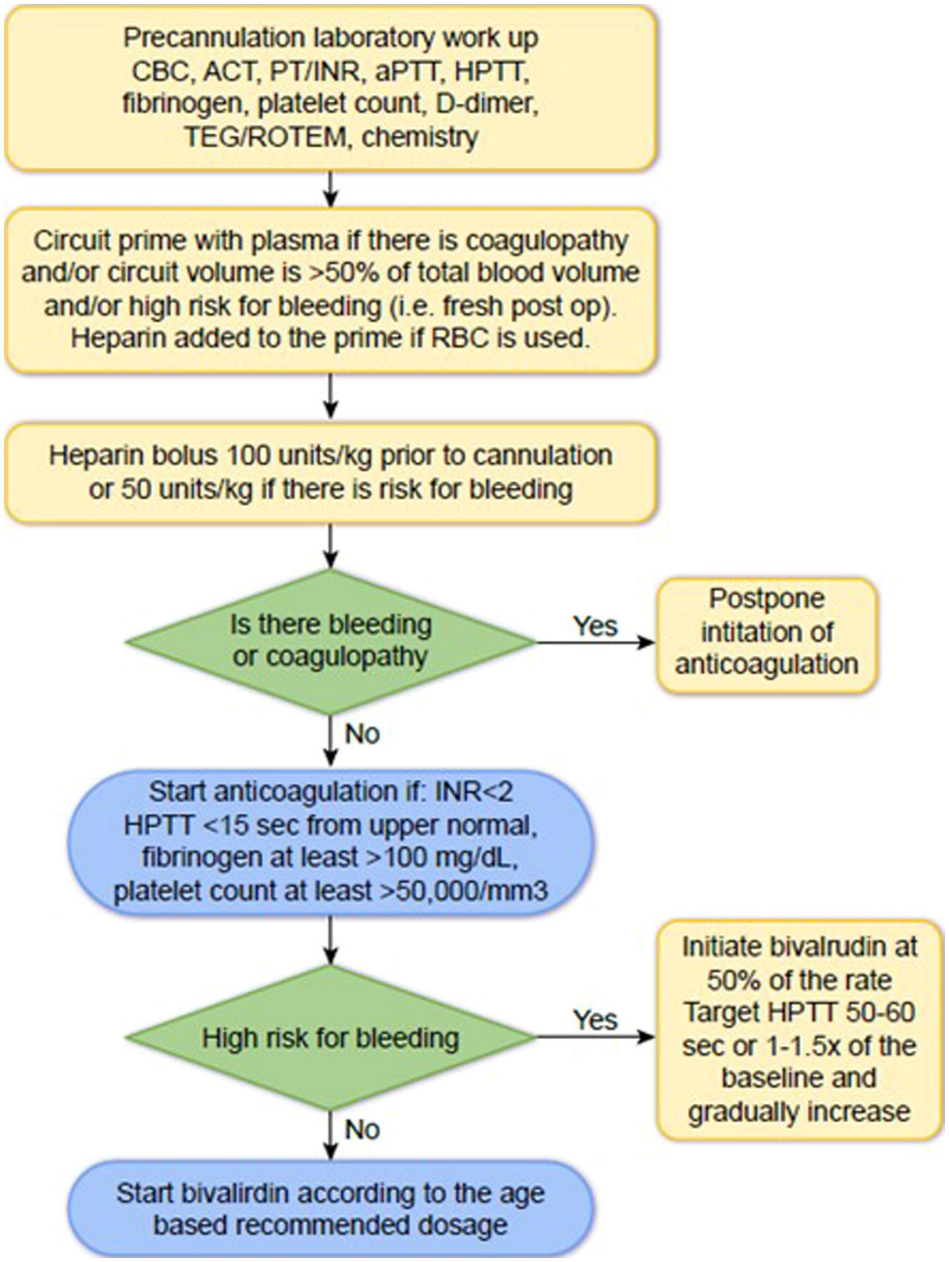
How to start bivalirudin at the initiation of ECMO CBC, complete blood count; ACT, activated clotting time; PT, prothrombin time; INR, international normalized ratio; aPTT, activated partial thromboplastin time; HPTT, aPTT with Hepzyme.

Bivalirudin can be started if ACT <200 s, INR <2.0, HPTT is within 15 s of the baseline, fibrinogen level is at least >100 mg/dL, and platelet count is at least >50,000/mm^3^ (Figure 2). Since ACT value depends on the instrument, it must be established at each hospital. If there is active bleeding or coagulopathy, practitioners should delay initiation of bivalirudin until bleeding is controlled and coagulopathy is resolved. The initial bivalirudin dosage can be adjusted based on creatinine clearance and patient age (Table 3).

**Table 3:** Bivalirudin initiation dose based on creatinine clearance and age.

Age-based initial dosage adjustment	Normal creatinine clearance for age (mg/kg/ h)	Mild–moderate decrease in creatinine clearance by age (mg/kg/ h)	Severely decreased creatinine clearance by age (mg/kg/ h)
Infants <1 year	0.3	0.2	0.1
Children 2–10 years	0.2	0.1	0.05
>10 years	0.15	0.1	0.05

#### Monitoring

The target HPTT is 2-3x baseline if there is no risk for bleeding, and 1.5-2x baseline if there is high risk for bleeding. HPTT should be repeated two hours after the initiation of bivalirudin and two hours after every titration (Table 4).

**Table 4:** Dose adjustment based on heparinase-treated aPTT.

Dose adjustment of bivalirudin using HPTT	Dose adjustment	Time to repeat HPTT
<60 s and/or < 1.5x from baseline	Increase infusion rate by 20%	2 h after dose adjustment
60–70 s and < 1.5x baseline	Increase infusion rate by 10%	2 h after dose adjustment
70–80 s and at least 1.5x baseline	No change	Repeat HPTT as per ECMO maintenance protocol
80–100 s and/or > 3x increase from baseline	Decrease infusion rate by 20%	2 h after dose adjustment
>100 s	Hold infusion for 1 h then restart at 50% less than previous rate	2 h after dose adjustment

HPTT, heparinase treated activated partial thromboplastin time; aPTT, activated partial thromboplastin time.

If dTT is available, the standard target is 0.8–2.6 μg/mL (46). Since a dTT assay is not standardized for ECMO anticoagulation, combining HPTT with dTT will help optimize the anticoagulation (Table 5). The target range for dTT may vary based on the assay and reagents, so each institution should adjust its protocol based on validation results. Target ranges reported in the literature are summarized in Table 6.

**Table 5:** Dose adjustment based on heparinase-treated aPTT and diluted thrombin time.

Dose adjustment of bivalirudin using dTT (bivalirudin level)	Dose adjustment	Time to repeat dTT and HPTT
<0.8 μg/mL and HPTT <60 s	Increase infusion rate by 20%	2 h after dose adjustment
1–2 μg/mL and HPTT <1.5x of baseline	Increase infusion rate by 10%	2 h after dose adjustment
1–2 μg/mL and HPTT >1.5x of baseline	No change	Repeat dTT as per ECMO maintenance protocol
>2.6 μg/mL	Decrease infusion rate by 20%	2 h after dose adjustment

dTT, diluted thrombin time; HPTT, heparinase-treated activated partial thromboplastin time; aPTT, activated partial thromboplastin time.

**Table 6:** Summary of reported dTT and aPTT target ranges in the literature.

	dTT target	aPTT target
Hasan et al., 2023 (36)	0.5–2.5 μg/mL	60–120 s
Teruya et al., 2021 (46)	0.8–2.6 μg/mL	1.7–2.3 x normal (60–80 s)
Beyer et al., 2020 (34)	0.5–1.5 μg/mL (dTT 40–80 s)	1.5–2 x normal (50–80 s)
Colucci et al., 2015 (47)	0.8–1.4 μg/mL	2.5–3.0 x normal (74–90 s)

dTT, diluted thrombin time; aPTT, activated partial thromboplastin time.

Follow-up laboratory studies, including PT/INR, aPTT/HPTT, dTT (if available), fibrinogen, platelet count, and D-dimer, should be repeated every six hours or four times a day. Frequency can be reduced once coagulation markers are stable and stable anticoagulation is achieved. ROTEM/TEG and PHb should be repeated daily (Table 7).

**Table 7:** ECMO coagulation panel and usual targets.

Test	Desired target/range	Purpose
INR	<2.5	To assess underlying coagulopathy
HPTT	60–80 s	Targeting bivalirudin anticoagulation
dTT (bivalirudin level)	0.8–2.6 μg/mL	
Fibrinogen	>200 mg/dL FEU	To monitor clot firmness
Platelet count	>100,000/mm^3^	
Plasma hemoglobin	<150 mg/dL	To monitor for hemolysis
D-dimer	Not established	To monitor fibrin formation and fibrinolysis in the circuit and patient’s circulation

INR, international normalized ratio; HPTT, heparinase-treated activated partial thromboplastin time; dTT, diluted thrombin time, FEU, fibrinogen equivalent unit.

#### Recently published review articles

There are several recent review articles comparing heparin to bivalirudin for ECMO anticoagulation (48–55), and all of them favor bivalirudin (Table 8). It should be noted, however, that all but one of these studies are meta-analyses and are compromised by the lack of standard definitions for bleeding and thrombosis. With this significant limitation in mind, the overall consensus favors bivalirudin for ECMO anticoagulation due to its potential ability to lower major bleeding risk, lower circuit and patient related thrombosis, and increase survival in both adult and pediatric patients.

**Table 8:** Review articles about heparin versus bivalirudin for ECMO anticoagulation.

Author, year	# of studies reviewed	Major findings
Li DH et al., 2022 (48)	9	Hospital mortality and thrombosis events decreased in adult patients on bivalirudin
Liu L et al., 2022 (49)	14	Risks of in-circuit and any thrombosis, hospital mortality, and bleeding are decreased on bivalirudin with positive impact on ECMO survival
Ma M et al., 2022 (50)	10	Major bleeding in children, adult patients’ thrombosis, in-circuit thrombosis/interventions and in-hospital mortality are reduced on bivalirudin
Li MJ, et al., 2022 (51)	9	Lower risks of major bleeding, ECMO in-circuit thrombosis, stroke and in-hospital mortality, and higher rates of survival to ECMO decannulation in patients on bivalirudin
Gu J et al., 2023 (52)	10	Reduction in thrombotic events, major bleeding events and in-hospital mortality in patients treated with bivalirudin
Wieruszewski PM et al., 2023 (53)	5	Higher risk of circuit-related thrombosis and to die in adult patients on heparin
Hasegawa D et al., 2023 (54)	11	Bivalirudin was associated with significantly lower short-term mortality
Valdes et al., 2023 (55)	7	Trends in lower rates of bleeding, transfusion requirements, and thrombosis with no difference in mortality in pediatric patients

All are meta-analysis except for Valdes et al. (55).

## Conclusion

According to several studies, bivalirudin shows a statistically significant advantage over heparin in preventing major bleeding and thrombosis, and in reducing transfusions. However, the majority of other studies showed no statistical difference between bivalirudin and heparin, likely due to the studies’ retrospective nature. Further prospective studies are needed to make a definitive determination.

## Author contributions

AN, VK, and JT contributed to conception of the review. AN and JT wrote the first draft of the review. VK made literature search, created tables, and edited final version. JT edited final version. All authors contributed to the article and approved the submitted version.

## Conflict of interest

The authors declare that the research was conducted in the absence of any commercial or financial relationships that could be construed as a potential conflict of interest.

## Publisher’s note

All claims expressed in this article are solely those of the authors and do not necessarily represent those of their affiliated organizations, or those of the publisher, the editors and the reviewers. Any product that may be evaluated in this article, or claim that may be made by its manufacturer, is not guaranteed or endorsed by the publisher.
